# Stroke Care Pathway ensures high-quality stroke management in the COVID-19 pandemic

**DOI:** 10.1038/s41598-023-32586-5

**Published:** 2023-04-05

**Authors:** Lukas Mayer-Suess, Annemieke ter Telgte, Silvia Praxmarer, Johann Willeit, Ewald Wöll, Theresa Geley, Heinrich Rinner, Michael Knoflach, Stefan Kiechl, The Tyrolean Stroke Pathway Group, Andreas Maurer, Adolf Schinnerl, Alexandra Rauter-Rzehak, Andreas Tür, Bernhard Oberwinkler, Christian Dengg, Patrick Loidl, Gudrun Schoenherr, Gudrun Seiwald, Hans Kreuzer, Hans-Robert Schoenherr, Heinrich Matzak, Heinrich Spiss, Hermann Kathrein, Hannes Gänzer, Johannes Schöch, Josef Grossmann, Julia Runge, Karin Willeit, Christian Boehme, Gerhard Klingenschmid, Thomas Toell, Raimund Pechlaner, Eva Hametner, Christoph Schmidauer, Martin Sojer, Klaus Berek, Klaus Engelhardt, Markus Mayr, Michael Baubin, Norbert Kaiser, Robert Perfler, Thomas Erlacher, Thomas Fluckinger, Wilhelm Grander

**Affiliations:** 1 Department of Neurology, Medical University of Innsbruck, Anichstraße 35, 6020 Innsbruck, Austria; 2 VASCage, Research Center on Vascular Ageing and Stroke, Anichstraße 5a, 6020 Innsbruck, Austria; 3 Tyrolean Health Care Fund, Eduard-Wallnöfer-Platz 3, 6020 Innsbruck, Austria; 4 Internal Medicine, Hospital St. Vinzenz, Sanatoriumstraße 43, 6511 Zams, Austria; 5 Emergency Medical Service, Innsbruck, Austria; 6 Hospital Schwaz, Schwaz, Austria; 7 Hospital Hochzirl, Zirl, Austria; 8 Doctors in Private Practice, Tyrol, Austria; 9 Hospital Reutte, Reutte, Austria; 10 Medical University Innsbruck, Innsbruck, Austria; 11 Tyrolean Health Care Fund, Innsbruck, Austria; 12 Hospital Lienz, Lienz, Austria; 13 Hospital Kufstein, Kufstein, Austria; 14 Department of Anesthesiology, Medical University Innsbruck, Innsbruck, Austria; 15 Hospital St Johann in Tirol, St Johann in Tirol, Austria; 16 Hospital Hall, Hall in Tirol, Austria

**Keywords:** Neuroscience, Neurology

## Abstract

The aim of our study was to assess whether a well-established federal state-wide Stroke Care Pathway delivering high quality stroke care can cope with the COVID-19 pandemic and associated measures to contain the virus spread. The retrospective analysis is based on a prospective, quality-controlled, population-based registry of all stroke patients in the Tyrol, a federal state of Austria and one of the early hot-spots of COVID-19 in Europe. Patient characteristics, pre-hospital management, intra-hospital management and post-hospital were analysed. All residents of the Tyrol suffering ischemic stroke in 2020 (n = 1160) and four pre-COVID-19 years (n = 4321) were evaluated. In 2020, the annual number of stroke patients was the highest in this population-based registry. When local hospitals were overwhelmed with SARS-CoV-2-patients, stroke subjects were temporarily allocated to the comprehensive stroke centre. Stroke severity, quality metrics of stroke management, serious complications, and post-stroke mortality did not differ between 2020 and the four comparator years. Notably, iv. thrombolysis-rate was similar (19.9% versus 17.4%, P = 0.25) and endovascular stroke treatment even better (5.9% versus 3.9%, P = 0.003) but resources for in-patient rehabilitation were limited (25.8% versus 29.8%, P = 0.009). Concluding, a well-established Stroke Care Pathway was able to maintain high-quality acute stroke care even when challenged by a global pandemic.

## Introduction

Since the WHO declared the coronavirus disease 2019 (COVID-19) a global pandemic on March 11th 2020, converging evidence suggests that stroke admissions have decreased and disruptions in the delivery of emergency stroke care, such as intravenous (iv.) thrombolysis, occurred^1–8^. Factors that propagated these changes have predominantly been attributed to efforts aimed to contain the spread of the virus^3,9–11^. Mortality has increased in COVID-19 times, in part because of impaired care of other diseases or, in case of ischemic stroke, due to the substantial reduction of acute stroke therapy^12^. To get an unbiased view of how COVID-19 affects stroke, reliable population-based data are mandatory. Our aim was to provide data from a federal state of Austria, that collects information of all stroke patients since 2009, and to investigate whether our well-established Tyrolean Stroke Pathway, that defines and continuously improves stroke management in the pre-hospital, intra-hospital, and rehabilitation field^13^, held up stroke-management-quality against one of the most strenuous crises in recent history.

## Methods

The following analyses were performed using data from the Tyrolean Stroke Pathway database, which provided one of the first evidence that Stroke Care Pathways improve patients’ outcome^13^. In short, the pathway was implemented in 2009, funded by the Tyrolean Government and health insurance carriers and has since been sustained to maintain high quality routine stroke care in the pre-hospital, hospital and post-stroke rehabilitation phase in the entire federal state. All patients that suffer ischemic- or hemorrhagic stroke and are admitted to any of the 8 hospitals in the Tyrol are included in the database. The subjects are identified through stroke discharge codes (International Statistical Classification of Diseases and Related Health Problems 10th Revision [ICD-10]) and digital data entry by a trained and qualified team is mandatory for reimbursement. Information on stroke characteristics (i.e. etiology, baseline National Institute of Health Stroke Scale [NIHSS]), pre-hospital management (i.e. emergency medical services transport modality), acute management timing and strategies (i.e. hospital arrival, acute imaging modalities, iv. thrombolysis and thrombectomy) in-house complications as well as early rehabilitation measures are supplemented by central governmental information about mortality, inter-hospital transfers discharge destinations and in-patient rehabilitation. Data collection and – entry into a three-page form, which is embedded within the electronic file of each stroke subject, was done by stroke teams located at each hospital in the Tyrol. Patients cannot be discharged from the treating hospital if the form is not completed. The database is monitored for completeness (100% complete) and accuracy by the Tyrolean Health Care Fund. The Stroke Care Pathway consists of more than 100 individual items, primarily focused on improving communication between sections (i.e. emergency medical service, hospital personnel, therapists) and organizational processes. Along with the documentation aspects, as previously reported^13^, the Tyrol Stroke Pathway encompasses the following selected key components: Repeated stroke awareness and information campaigns (especially focused on the general public), prehospital stroke codes, hospital pre-notification, prehospital triage algorithm, algorithms for helicopter and emergency doctor involvement, standardized information transfer, practice guidelines for diagnostic work-up and stroke therapy, thrombolysis administration protocol, early routine mobilization of patients, practice standards for nurses and therapists, assembly of stroke teams, continuous educational activities for all health care professions, obligatory NIHSS training, CT prioritization, obligatory dysphagia testing, standardized assessment of stroke complications, tele-radiology, early state-of-the-art secondary prevention, standardized discharge management, 24/7 access to stroke expertise, competence platform and decision support, web-based stroke pathway, regional rehabilitation transfer agreements, emergency medical service bypass and referral protocols, annual feedback visits, continuous feedback of achievements, standardized electronic documentation of selected quality parameters, education of and educational material for patients and their relatives, follow-up at three months, therapy standards for inpatient rehabilitation, network for quality-controlled outpatient rehabilitation.

### Patient recruitment and selection

In all, only patients coded as I63 as a main diagnosis were considered for the analysis. ICD 10 Codes I62, I64 and G46 are not permitted for reimbursement by the Tyrolean Healthcare Fund and source data verification of G45 coded patients are regularly performed by the data management team to ensure delimitability between ischemic stroke and TIA patients. All residents of the Tyrol suffering ischemic stroke treated in any Tyrolean hospital in 2015–2017, 2019 and 2020 were included in the study. 2020 was considered the COVID-19 year whilst all others (2015–2017 and 2019) were pre-COVID-19 comparators^14^. Based on database maintenance, data input was disabled for 3 months in 2018, therefore 2018 was not considered in the current analysis. The Tyrol has high mountain chains along its borders to Italy and Germany and, accordingly, the proportion of acute stroke patients living within the catchment area but being treated outside the Tyrol was well below 1.0% per year, thus negligible, (data source Documentation and Information Systems for Analyses in Health Care [DIAC]) and the hospitalization rates for stroke was 97.4%^13^. There was no formal patient involvement plan during the set-up process of the Tyrol Stroke Pathway.

### Statistical methodology

Statistical analyses were performed using R, version 4.2.2 (R-Core Team [2021]. R-Foundation for Statistical Computing, Vienna, Austria). Confidence intervals for proportions were obtained by the Clopper-Pearson method. Chi-squared test for categorical and Mann–Whitney-U test for continuous variables examined group differences.

### Standard protocol approvals, registration, patient consents

Analyses were approved by the local ethics committee at the Medical University of Innsbruck (EK#1152/2020). Data was collected as part of the governmental quality-assurance dataset of the Tyrolian Stroke Pathway based on the Tyrolean Healthcare Fund law (TGFG §18) and the federal law on health care documentation and the target control health (Art. 15a Bundesverfassungsgesetz–Zielsteuerung Gesundheit). Therefore, individual patient consent was not required. All methods were carried out in accordance with relevant guidelines and regulations. Anonymized data not published within this article will be made available by request from any qualified investigator.

## Results

**Figure 1:**
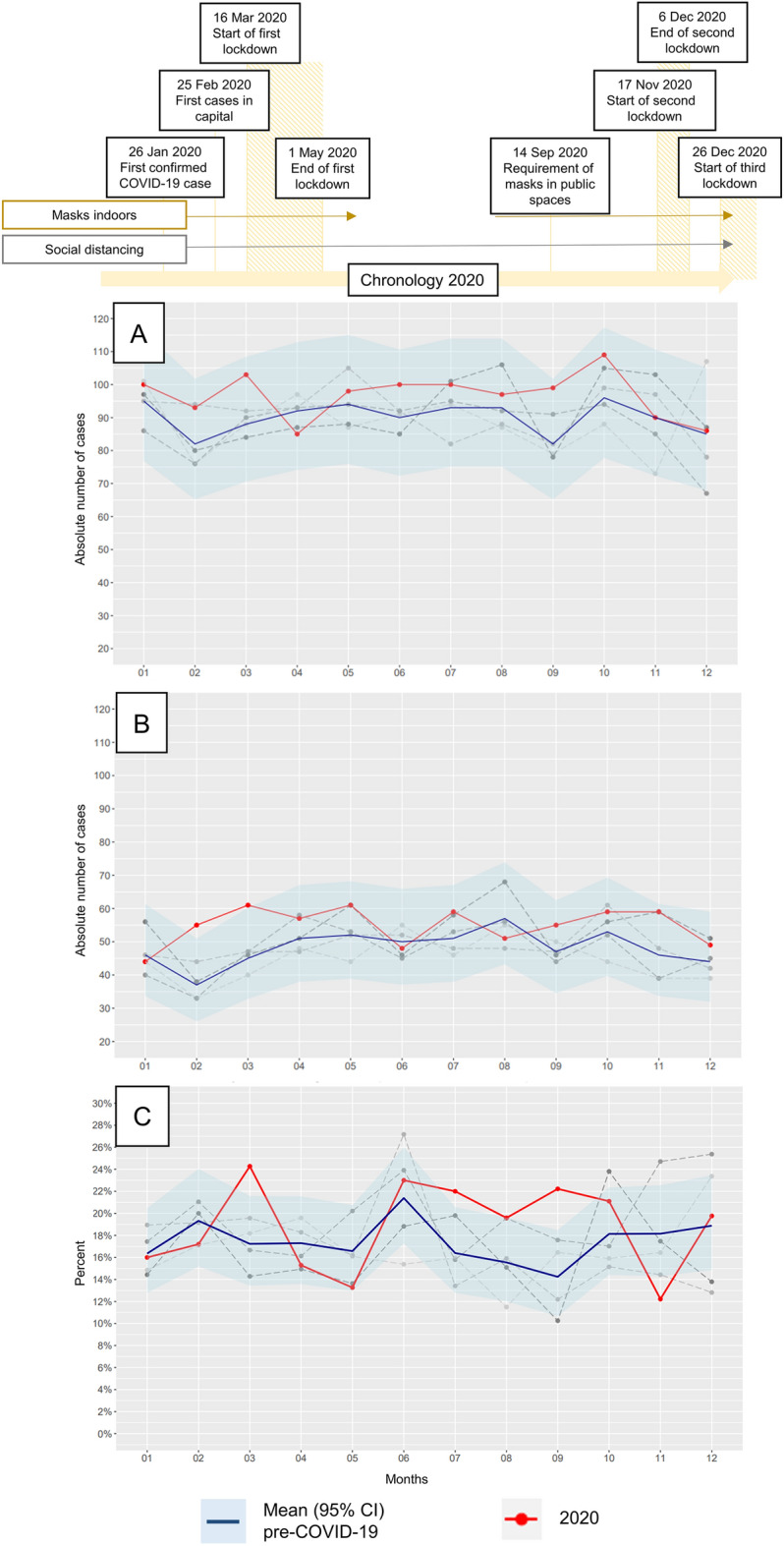
Chronology of COVID-19-measures and absolute number per month of stroke admissions (**A**), minor strokes (NIHSS ≤ 4) (**B**) as well as of rate per month of stroke patients with receiving i.v. thrombolysis (**C**).

Baseline characteristics of 5481 stroke patients (n = 1160 in COVID-19 and n = 4321 in the four comparator years) are given in Table 1 and Fig. 1.

**Table 1:** Differences between patient characteristics and pre-/intra-hospital- and post-hospital-stroke care metrics comparing pre-COVID-19 years and 2020.

	Controls (2015, ’16, ’17, ’19)	COVID-19 year (2020)	P-value
Baseline characteristics			
N	4321	1160	
Female^#^	1994 (46.1; 44.7–47.6)	504 (43.4; 40.6–46.4)	0.11
Age*	76 (66–83)	75 (65–82)	0.06
NIHSS*	3 (1–8)	3 (1–7)	0.12
Pre-hospital phase			
Direct admission to stroke center^#^	2263 (52.4; 50.9–53.9)	660 (56.9; 54.0–59.8)	0.007
Secondary transfer to stroke center^#^	344 (8.0; 7.2–8.8)	90 (7.8; 6.3–9.5)	0.87
Helicopter transport^#^	208 (4.8; 4.2–5.5)	63 (5.4; 4.2–6.9)	0.43
Symptom to door ≤ 3 h^#^	1621 (37.5; 36.1–39.0)	417 (35.9; 33.2–38.8)	0.34
Onset unknown^#^	1543 (35.7; 34.3–37.2)	401 (34.6; 31.8–37.4)	0.49
Hospital phase			
Cerebral imaging ≤ 1 h^#^	3292 (77.1; 75.8–78.4)	888 (77.8; 75.3–80.2)	0.65
Iv. Thrombolysis^#^	754 (17.4; 16.3–18.6)	220 (19.0; 16.7–21.3)	0.25
Door to needle time*	45 (28–72)	42 (28–68)	0.95
Thrombectomy^#^	160 (3.7; 3.2–4.3)	62 (5.3; 4.1–6.8)	0.014
Stroke unit care^#^	2643 (61.2; 59.7–62.6)	708 (61.0; 58.2–63.9)	0.96
Intensive care^#^	415 (9.6; 8.7–10.5)	116 (10.6; 8.3–11.9)	0.73
Length of hospital stay*	9 (6–14)	9 (6–13)	0.28
Severe complication^#^	317 (7.3; 6.6–8.2)	98 (8.4; 6.9–10.2)	0.23
Urinary tract infection^#^	80 (1.9; 1.5–2.3)	42 (3.6; 2.6–4.9)	< 0.001
In-house mortality^#^	6.7 (5.9–7.5)	5.9 (4.6–7.4)	0.36
Post-hospital phase			
In-patient rehabilitation^#^	29.8 (28.4–31.1)	25.8 (23.3–28.4)	0.01

^#^Values given as N (%; 95% CI).

*Values given as median (IQR).

Severe complications composite variable: infections (pneumonia, sepsis), myocardial infarction, recurrent or progressive stroke, intracranial- or extracranial bleeding, epileptic seizure and deep vein thrombosis or pulmonary embolism.

Cerebral imaging ≤ 1 h: cerebral imaging (CT/MRI) within 1 h after hospital admission.

In 2020, the annual number of stroke patients was the highest in our population-based registry which is active from 2009 onwards. Stroke incidence was 153.1/100.000 compared to 145.5/100.000 in the four comparator years, which was not statistically significant (P = 0.13). There were no differences in stroke severity, relevant quality indices of acute stroke management or post-stroke mortality. In 2020, however, stroke patients were more frequently allocated to the comprehensive stroke center, rate of endovascular thrombectomy increased and post-stroke care in rehabilitation centers decreased. Concerning in-hospital treatment, severe complications were unchanged but urinary tract infections in 2020 were more frequent.

## Discussion

Cardiovascular disease is the main cause of death, with stroke being the most frequent, devastating, time-critical vascular emergency and one of the leading causes of disability world-wide^13,15^. Therefore, even in challenging times, it is indispensable to provide quality care for stroke subjects. Stroke is a serious complication of COVID-19 affecting approximating 1.3% of all cases^16^. Previous evidence suggested that the COVID-19 pandemic has impacted stroke care around the globe with decreased hospital admissions, especially of minor stroke patients, and a decline in rates of iv. thrombolysis and endovascular thrombectomy^1,3,6,7^. We report the impact of COVID-19 in a federal state of Austria using a population-based registry of near 100% of all stroke patients in the region. The Tyrol was an early hot-spots of COVID-19 in Europe. Internal and neurological hospitals treating stroke patients were crucially involved in managing severe acute respiratory syndrome coronavirus type 2 (SARS-CoV-2) patients but stroke units and angiosuites were preserved. The annual number of stroke patients in 2020 was the highest in our population-based registry (active from 2009 onwards). Stroke severity (including minor stroke) and stroke characteristics remained unchanged (Fig. 1). Previous studies have reported a decline in stroke admissions and acute management strategies such as i.v. thrombolysis especially in non-stroke centers (drip-and-ship concept) during COVID-19^17,18^. The Tyrolean Stroke Pathway recommends the mothership concept for patients with severe stroke symptoms if the thrombectomy center can be reached within 30 min. Accordingly, during the COVID-19 pandemic, hospital allocation of stroke patients has temporarily changed, whenever local hospitals were overwhelmed with SARS-CoV-2 patients and stroke patients were directly transported to the comprehensive stroke center Innsbruck (mothership)^17,18^. In spite of the measures taken in the whole federal state to prevent intra-hospital COVID-19 spread, the rate of iv. thrombolysis was at least as good as in the four comparator years (Table 1, Fig. 1). Further, time-dependent quality indices of acute stroke management remained unchanged with rates of patients being admitted within 3 h of symptom onset and subjects having initial cerebral imaging within 1 h of hospital admission as well as door-to-needle-time seeing no significant difference (Table 1). The rate of thrombectomy has annually increased since the landmark trials of 2015 (thrombectomy rates 2015, 2016, 2017 and 2019 were 2.7%, 3.6%, 3.9%, and 4.6%) and the up-wards trajectory persists in 2020 (5.3%, P = 0.014)^19^. This increase might in-part be due to a higher incidence of large vessel occlusions reported in literature during the COVID-19 pandemic^20,21^. No change emerged concerning serious in-house complications and stroke mortality. Urinary tract infections were more frequent in 2020 compared to previous years, which may be due to increased screening for sources of fever and inflammation in COVID-19 times. This finding is in line with previous reports suggesting higher rates of infections overall in rehabilitation eligible patients unable to be transferred to rehabilitation facilities^22^. As one of the two stroke rehabilitation centers in Tyrol was partially reassigned to treat SARS-CoV-2 patients in early spring and late autumn in 2020, the share of in-patient post-stroke rehabilitation was impaired (25.9% versus 29.8%) and partly compensated by out-patients rehabilitation. In conclusion, the Tyrol Stroke Pathway, through its network of stake-holders making timely decisions and its continuous stroke awareness campaigns to Tyrolian inhabitants, was able to maintain high-quality stroke care even when challenged by a global pandemic. Therefore, we emphasize on the positive impact that structured treatment pathways have on the functional outcome of ischemic stroke subjects, which could presumably be extrapolated to other time-critical emergencies, such as myocardial infarction. The key strength of our study is the Tyrol Stroke Pathway database being one of the very few population-based registries that covers information on all stroke patients in a federal state irrespective of the treating hospital, which additionally has full data monitoring and no missing data.

## Data Availability

The datasets used and/or analysed during the current study available from the corresponding author on reasonable request.
